# Extranodal Natural Killer (NK)/T-Cell Lymphoma Causing Severe Facial Destruction Refractory to Chemotherapy and Immunotherapy

**DOI:** 10.7759/cureus.104253

**Published:** 2026-02-25

**Authors:** Ryan A Tiu, Jonathan F Garcia

**Affiliations:** 1 Internal Medicine, University of California Los Angeles David Geffen School of Medicine, Los Angeles, USA

**Keywords:** ebv-associated lymphoma, epstein-barr virus, extranodal natural killer/t-cell lymphoma, nasal type, non-hodgkin’s lymphomas, pembrolizumab with chemotherapy

## Abstract

Extranodal natural killer/T-cell lymphoma, nasal type (ENKTCL), is a rare, highly aggressive Epstein-Barr virus (EBV)-associated non-Hodgkin lymphoma. Diagnosis is frequently delayed because early presentation is often non-specific and may mimic many other conditions, including disseminated fungal or atypical bacterial infections, autoimmune conditions such as vasculitis, and other cutaneous malignancies, such as peripheral T-cell lymphomas or melanoma. We present a case of extensive, rapidly progressive and highly destructive necrotic facial and cutaneous lesions, which remained undifferentiated despite initial diagnostic workup, necessitating travel to a quaternary medical center on a medical visa for further evaluation. A thorough workup, including a histopathologic evaluation along with a positive EBV-encoded RNA in situ hybridization, confirmed ENKTCL. The patient’s clinical course was marked by extensive local tissue destruction, EBV viremia, severe malnutrition, recurrent episodes of shock, and disease progression despite treatment with pegaspargase, gemcitabine, and oxaliplatin (P-GEMOX) and modified dexamethasone, methotrexate, ifosfamide, L-asparaginase, and etoposide (SMILE) chemotherapy and pembrolizumab. This case underscores the diagnostic challenges of ENKTCL and highlights the aggressive clinical course associated with delayed diagnosis and advanced-stage disease, emphasizing the importance of early histopathologic evaluation with EBV testing in patients with progressive necrotic midline lesions.

## Introduction

Natural killer (NK) cells are cytolytic immune cells that secrete granules containing perforin and granzymes. Extranodal natural killer/T-cell lymphoma, nasal type (ENKTCL) is a rare and highly aggressive non-Hodgkin lymphoma associated with Epstein-Barr virus (EBV) infection [1]. ENKTCL demonstrates the highest prevalence in Asia and South America with all affected groups exhibiting EBV infection, and a median age at diagnosis ranging from 46 to 52 years [2-4]. ENKTCL is the third most common subtype of peripheral T-cell lymphoma in Latin America, accounting for 15% of peripheral T-cell lymphoma cases overall and 31% of cases in Mexico [3].

ENKTCL primarily affects the nasal cavity and upper aerodigestive tract, but can rapidly disseminate to the skin, gastrointestinal tract, and lymph nodes. Patients typically present with nasal obstruction followed by discharge, epistaxis, and necrotic ulceration secondary to vascular damage [2]. Forty percent of patients also report B symptoms, such as fevers, drenching night sweats, or unintentional weight loss [5]. Diagnosis is supported by histopathologic evaluation with immunohistochemistry positive for cytoplasmic CD3, CD56, and the cytotoxic molecules perforin, granzyme B, and T-cell intracellular antigen-1 (TIA-1). Definitive diagnosis also requires positive EBV in situ hybridization [4].

Early diagnosis of ENKTCL remains challenging as its initial presentation frequently mimics inflammatory, infectious, or alternative malignant processes. We report a case of ENKTCL in a young woman from Mexico requiring international consultation to arrive at the correct diagnosis. Her disease proved refractory to three lines of therapy, with her course complicated by severe malnutrition and recurrent episodes of shock, exemplifying the aggressive nature of this malignancy and the clinical consequences of delayed diagnosis.

## Case presentation

A 34-year-old woman with no significant past medical history presented for evaluation of a progressive, painful facial lesion. Over six months, the lesion rapidly evolved from a localized black, purulent, bleeding ulcer on the left cheek to progressively involve the forehead, eyes, nasal cavity, and lips. Cutaneous disease was accompanied by malaise, intermittent fevers, and epistaxis. One month prior to presentation, she noticed the development of a similar lesion on her right foot. 

Multiple evaluations in Mexico, including tissue histopathology and culture, were non-diagnostic and left a broad differential diagnosis, including infections, such as disseminated mycoses or atypical infections such as cutaneous anthrax or disseminated tuberculosis; autoimmune or inflammatory etiologies such as vasculitis; or other cutaneous malignancies such as other T-cell lymphomas or melanoma. The only treatment she had received was an unknown course of antibiotics. The patient then sought care in the United States on a medical visa. 

**Figure 1:**
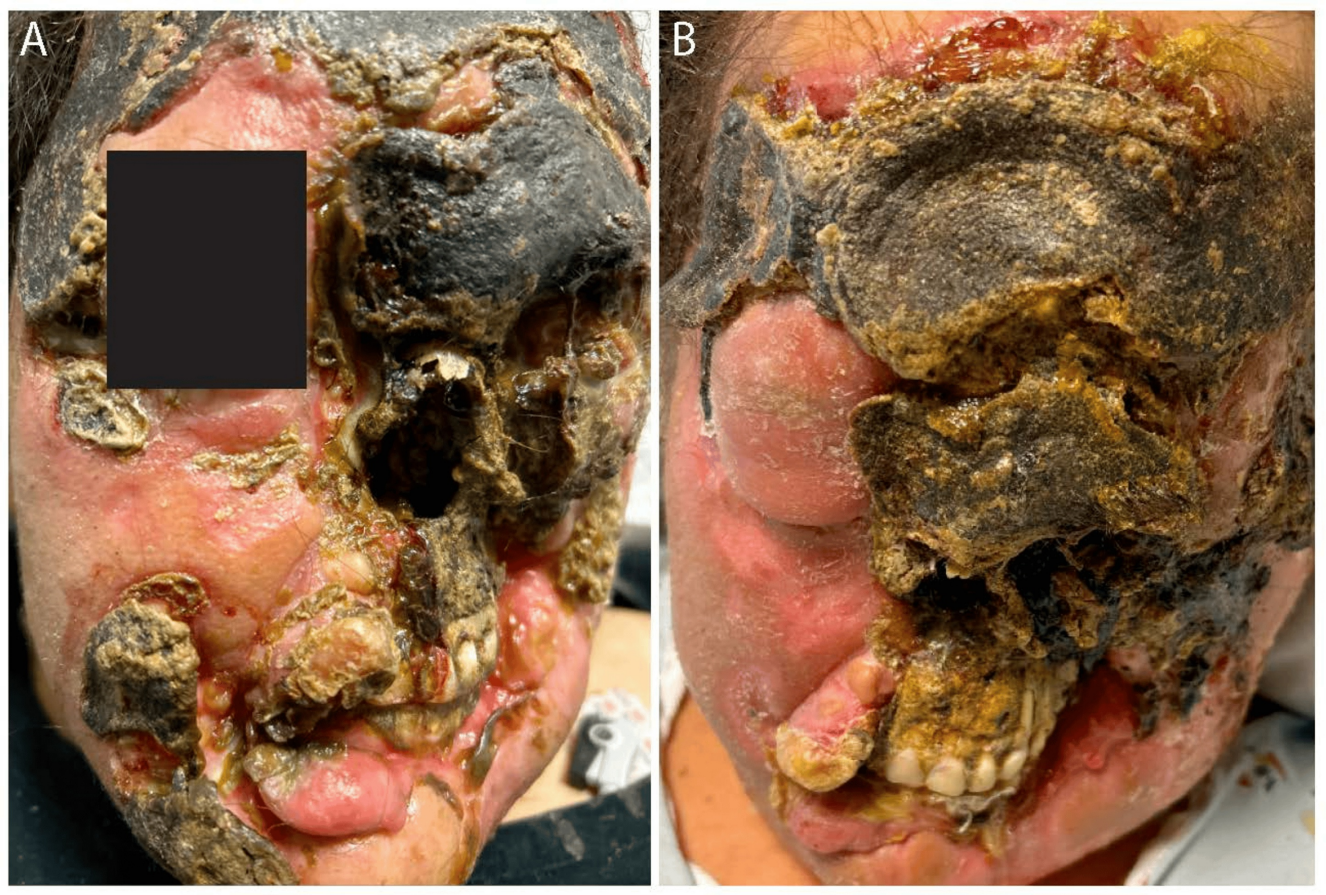
Facial lesions at admission (A) and two months later following treatment with P-GEMOX, modified SMILE, and pembrolizumab (B). P-GEMOX: pegaspargase, gemcitabine, and oxaliplatin; SMILE: dexamethasone, methotrexate, ifosfamide, L-asparaginase, and etoposide.

The patient was hemodynamically stable on admission. Labs were notable for macrocytic anemia, elevated ferritin and lactate dehydrogenase (LDH), hyponatremia, hypokalemia, hypocalcemia, and folate deficiency (Table 1). She was found to have EBV viremia with EBV DNA quantitative polymerase chain reaction (PCR) of 9,432 IU/mL. Physical examination revealed extensive erosion of the left eyelid causing complete left-eye vision loss. Erosion of the external nose exposed the nasal cavity (Figure 1). Five eschar-like lesions were present on the lower extremities.

**Table 1:** Laboratory findings at hospital admission, immediately after cycle one P-GEMOX chemotherapy, and two months into admission. EBV: Epstein-Barr virus; P-GEMOX: pegaspargase, gemcitabine, and oxaliplatin.

Test	Reference range	At admission	After cycle one P-GEMOX chemotherapy	Two months into admission
White blood cell count (x10^3^/uL)	4.16-9.95	4.78	3.15	3.36
Hemoglobin (g/dL)	11.6-15.2	6.3	8.3	8
Mean corpuscular volume (fL)	79.3-98.6	103.3	92.8	90.8
Ferritin (ng/mL)	8-180	1,859	5,304	4,479
Sodium (mmol/L)	135-146	127	147	134
Potassium (mmol/L)	3.6-5.3	3.4	3.3	4.1
Calcium (mg/dL)	8.6-10.4	7.7	8.1	8.2
Folate (ng/mL)	8.1-30.4	4.7	>40.0	Not checked
Alanine transaminase (U/L)	8-70	13	609	242
Aspartate transaminase (U/L)	13-62	24	453	403
Alkaline phosphatase (U/L)	37-113	151	341	430
Lactate dehydrogenase (U/L)	125-256	282	350	534
EBV DNA quantitative PCR (IU/mL)	Not detected	9,432	5,030	986

**Figure 2:**
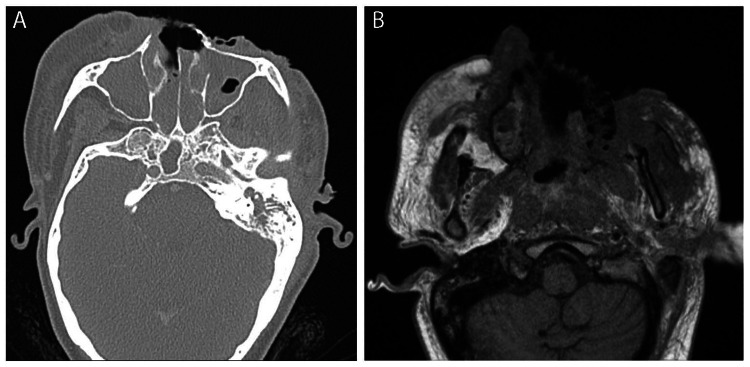
(A) Axial CT at admission showing severe multifocal paranasal sinus opacification with osseous erosions and soft tissue ulceration overlying the anterior and left lateral face with bilateral retroantral, retropharyngeal, and retro-orbital extension. (B) Axial T1-weighted MRI at admission showing extensive soft tissue edema and enhancement with deep overlying ulcerations in the left paramedian face.

CT of the neck, brain, face, and sinus (Figure 2A) and MRI of the face and orbits (Figure 2B) demonstrated osseous erosions and soft tissue ulceration of the left face with bilateral retroantral, retropharyngeal, and retro-orbital extension. CT of the chest and abdomen did not demonstrate metastatic disease. Given persistent fevers, empiric broad-spectrum antibiotics and antifungals were started. The patient's history of sheep exposure, geographic region of origin within Mexico, and eschar-appearing lesions prompted a PCR test for cutaneous anthrax, which was negative. Wound cultures grew methicillin-sensitive *Staphylococcus aureus* (MSSA), *Enterobacter*, and *Pseudomonas*.

Thigh skin biopsy revealed ENKTCL positive for CD2, CD3, T-cell receptor (TCR) gamma, TIA-1, and granzyme B, with positive EBV-encoded RNA in situ hybridization. Given the advanced stage of her disease, she was started on systemic treatment with pegaspargase, gemcitabine, and oxaliplatin (P-GEMOX) chemotherapy. Fluoroscopy-guided lumbar puncture was performed to assess for central nervous system involvement, and intrathecal methotrexate was administered. Flow cytometry of the cerebrospinal fluid (CSF) was negative for increased or abnormal T/NK cells. CSF cytology was negative for malignant cells. At the end of the first cycle of chemotherapy, the patient’s EBV DNA level was 5,030 IU/mL (Table 1).

The patient's hospital course was complicated by multiple intensive care unit admissions for multifactorial shock attributed to poor oral intake, insensible losses, and recurrent soft tissue infection and MSSA bacteremia. Inability to tolerate oral intake from severe facial involvement and trismus led to profound malnutrition, requiring total parenteral nutrition and surgical gastrostomy tube placement. Active eschar sloughing precluded surgical debridement.

**Figure 3:**
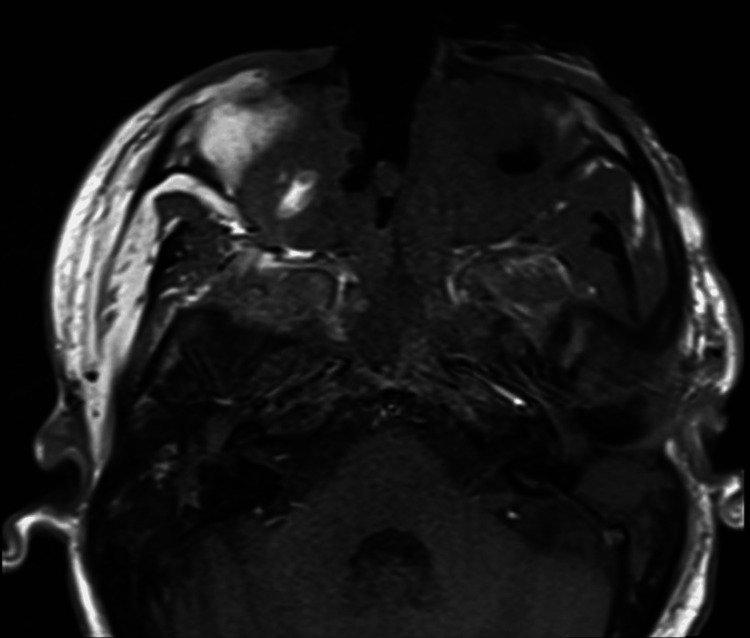
Axial T1-weighted MRI of the face and orbits two months into admission. Shown are stable orbital imaging and redemonstration of extensive deep, heterogeneously enhancing ulcerations of the face with osseous destruction of the facial bones and nasal cavity.

Two weeks into hospitalization, the patient underwent further treatment with pembrolizumab and modified SMILE chemotherapy without pegaspargase due to presumed pegaspargase-induced liver injury from a prior dose. Despite having tried three lines of therapy, her disease continued to progress. An interval MRI of the face and orbits two months after admission was notable for progressive ulceration and osseous destruction of the face (Figure 3).

Given the refractory nature of her disease, worsening functional status, severe malnutrition, and no additional treatment options, the patient elected to transition to comfort care. The patient was transported to Mexico via air medical transport for hospice care in accordance with her wishes.

## Discussion

Diagnosis of ENKTCL is frequently delayed due to its non-specific initial presentation and misdiagnosis as an infectious or inflammatory disease, often resulting in empiric antibiotic treatment. The mean interval from symptom onset to diagnosis is approximately 4.8 months [4]. In the present case, early non-specific constitutional symptoms preceded the development of edema and necrotic ulceration, contributing to a significant diagnostic delay.

When identified in the early stage (stage I/II), localized nasal ENKTCL can be treated with radiotherapy and concurrent or sequential chemotherapy, an approach that has led to improved outcomes in recent years. Reported five-year overall survival (OS) rates range from 40% to 70% [1,2,4,6-8], though long-term survival remains poor at less than 40% [5]. Outcomes from cyclophosphamide, doxorubicin, vincristine, and prednisone (CHOP) chemotherapy alone are poor (five-year OS 0% to 34%) due to tumor cell expression of P-glycoprotein, which mediates efflux pump action to confer multidrug resistance to anthracycline-based regimens [2].

Advanced-stage, relapsed, and refractory ENKTCL are associated with worse prognosis, with five-year OS rates of 24% to 41% [7,8]. SMILE therapy or P-GEMOX is recommended as induction therapy, with comparable efficacy [9]. Preferred chemotherapy varies by institution. In this case, P-GEMOX was initially utilized given a slightly more favorable toxicity profile than SMILE. The utility of hematopoietic stem cell transplantation remains under investigation; however, retrospective studies suggest that patients with advanced or refractory ENKTCL may achieve durable remission following allogenic hematopoietic stem cell transplantation after chemotherapy [2,10]. Pembrolizumab, a monoclonal antibody against the programmed death 1 receptor (PD-1), is also under further investigation for the treatment of relapsed or refractory ENKTCL and has shown efficacy for some patients who achieved complete response and durable remission [11,12].

Given the heterogeneity of outcomes in ENKTCL, multiple prognostic indices have been developed. The International Prognostic Index (IPI), widely used in B-cell lymphomas, incorporates age, performance status, extranodal involvement, stage, and serum LDH. However, the IPI’s prognostic utility in ENKTCL has been inconsistent. While several studies in Latin American and East Asian cohorts failed to demonstrate prognostic value [6,13-15], others reported meaningful risk stratification [5,16]. Consequently, alternative models, including the NK Prognostic Index, Korean Index, and Prognostic Index of Natural Killer Lymphoma (PINK), have been proposed. These indices emphasize advanced stage (stage III/IV, median OS six months compared with 90 months in non-advanced disease [17]), performance status (Eastern Cooperative Oncology Group (ECOG) performance status >2), presence of B symptoms, elevated serum LDH, and lymph node involvement.

In this case, treatment with pembrolizumab was attempted in the setting of the patient’s advanced-stage disease refractory to chemotherapy; however, even pembrolizumab proved ineffective. This is consistent with her multiple adverse prognostic features, including advanced-stage disease, B symptoms, elevated serum LDH, regional lymph node involvement, and poor performance status.

This patient also had an active EBV infection as evidenced by positive serum titers. EBV plays a central role in the pathogenesis and prognosis of ENKTCL. In chronic active EBV infection, infected T/NK cells can accumulate genomic alterations that promote malignant transformation [4]. In addition, EBV-infected NK cells secrete cytokines that enhance cellular proliferation and upregulate the oncogenic protein latent membrane protein 1 (LMP1) [4]. As lymphoma cells undergo apoptosis, EBV DNA is released into the circulation and can serve as a biomarker of tumor burden [18]. Elevated pretreatment EBV DNA levels are associated with the previously discussed clinical features of B symptoms, elevated LDH, worse performance status, and high IPI score, whereas patients with pretreatment EBV DNA levels ≤500 copies/mL demonstrate improved treatment response and survival. Moreover, detectable EBV DNA following treatment completion strongly predicts poorer outcomes and relapse, with reported three-year OS rates of 41% compared with 92% in patients with undetectable viral load [19]. 

These observations are incorporated into the PINK with EBV DNA (PINK-E), which stratifies patients into low-, intermediate-, and high-risk groups corresponding with three-year OS rates of 81%, 55%, and 28%, respectively [20]. Accordingly, quantitative EBV DNA measurement should be performed at diagnosis and monitored longitudinally during treatment and follow-up.

## Conclusions

ENKTCL is a rare, highly aggressive malignancy whose early manifestations can mimic infectious or inflammatory disease, potentially leading to significant diagnostic delay. Notably, a definitive diagnosis was not achieved in this case despite multiple evaluations in Mexico, reflecting challenges in access to care. This case illustrates clinical consequences of delayed recognition, including extensive soft tissue destruction, systemic complications, and progression to refractory disease. The presence of advanced-stage disease, multiple adverse prognostic features, and elevated EBV viral load was consistent with the poor clinical outcome. Early consideration of ENKTCL and prompt histopathologic evaluation with EBV testing in patients presenting with progressive necrotic midline lesions remains essential to improving outcomes in this otherwise devastating disease.
